# Coordinated Activity of Transcriptional Networks Responding to the Pattern of Action Potential Firing in Neurons

**DOI:** 10.3390/genes10100754

**Published:** 2019-09-26

**Authors:** Dumitru A. Iacobas, Sanda Iacobas, Philip R. Lee, Jonathan E. Cohen, R. Douglas Fields

**Affiliations:** 1 Personalized Genomics Laboratory, Center for Computational Systems Biology, Prairie View A&M University, Prairie View, TX 77446, USA; daiacobas@pvamu.edu; 2 DP Purpura Department of Neuroscience, Albert Einstein College of Medicine, Bronx, NY 10461, USA; 3 Department of Pathology, New York Medical College, Valhalla, NY 10595, USA; sandaiacobas@gmail.com; 4 Section on Nervous System Development and Plasticity, the *Eunice Kennedy Shriver* National Institute of Child Health and Human Development (NICHD), NIH, Bethesda, MD 20892, USA; leeph@mail.nih.gov; 5 Division of Medical Imaging Products, U.S. Food and Drug Administration, Silver Spring, 20993 MD, USA; electronlove@gmail.com

**Keywords:** calcium voltage-dependent channels, calcium signaling, covariance analysis, DRG neurons, gene expression variability, potassium voltage-gated channels, sodium voltage-gated channels, transcription factors

## Abstract

Transcriptional responses to the appropriate temporal pattern of action potential firing are essential for long-term adaption of neuronal properties to the functional activity of neural circuits and environmental experience. However, standard transcriptome analysis methods can be too limited in identifying critical aspects that coordinate temporal coding of action potential firing with transcriptome response. A Pearson correlation analysis was applied to determine how pairs of genes in the mouse dorsal root ganglion (DRG) neurons are coordinately expressed in response to stimulation producing the same number of action potentials by two different temporal patterns. Analysis of 4728 distinct gene-pairs related to calcium signaling, 435,711 pairs of transcription factors, 820 pairs of voltage-gated ion channels, and 86,862 pairs of calcium signaling genes with transcription factors indicated that genes become coordinately activated by distinct action potential firing patterns and this depends on the duration of stimulation. Moreover, a measure of expression variance revealed that the control of transcripts abundances is sensitive to the pattern of stimulation. Thus, action potentials impact intracellular signaling and the transcriptome in dynamic manner that not only alter gene expression levels significantly (as previously reported) but also affects the control of their expression fluctuations and profoundly remodel the transcriptional networks.

## 1. Introduction

Expression of the 20,000–25,000 protein-coding genes in mammalian neurons is regulated by a complex, inter-coordinated network that is constantly in a dynamic state of modulation influenced by neural impulse activity. The most common method of analyzing gene transcription in cells is by measuring the abundance of tens of thousands of specific gene transcripts simultaneously in cells by microarray or RNA sequencing. The values obtained from multiple biological replicate experiments are represented by a single measure of central tendency of gene expression level for each gene product, typically the arithmetic mean. Statistical comparisons are made to identify mRNA transcripts that are significantly increased or decreased in abundance following an experimental treatment relative to a control. Although this approach is valid and insightful, it is limited in determining how transcriptional networks may be modulated in response to an external stimulus, especially so when the stimulus applied is temporally dynamic and fails to drive the cell into a steady state. Standard analysis also ignores changes in the control of transcripts abundances and in the interplay of functional pathways caused by the experimental treatment.

This is the situation in neurons responding to action potential input of different temporal patterns within the physiological range of activation. Under normal conditions, transcriptional networks are being dynamically activated (or inhibited) by action potential firing. However, this activity may fail to produce a measurable increase or decrease in abundance of a given mRNA transcript, even though the transcript is part of a transcriptional network that is being modulated dynamically. This may occur because, in contrast to receptor–ligand-mediated signaling in most non-neuronal cells, neuronal response to sensory input, and information processing within neural networks, is a temporal code of transient membrane depolarizations. In neurons, the action potential firing frequency and burst patterns, together with synchrony and phase relationships among populations of interconnected neurons, is the fundamental mechanism of operation [1]. Neurons cannot process or transmit information unless they are maintained in homeostatic balance. Many cellular and network-level interactions dynamically regulate neuronal excitability to keep the neuron near a setpoint of homeostatic balance to achieve optimal information processing [1].

Analysis of only the expression level of individual genes fails to exploit the richer information available from the microarray and sequencing data which quantifies the expression levels of all genes simultaneously, beyond what could have been obtained by measuring each gene separately. By analogy, measuring volume of traffic of different types of vehicles on roads would be insufficient to elucidate the complex structure and operation of a transportation network; how the traffic is coordinately changing is an essential feature. A convoy is not typically recognized by identifying vehicles exceeding the speed limit, but rather by the highly coordinated changes in speed among all the vehicles in the convoy. Likewise, the critical aspect of gene network function is the coordinated activity of elements in the network. 

Various forms of (weighted and unweighted) correlation network analyses were developed and widely used in the genomics field (e.g., [2,3,4,5]) to reveal the underlying structure of “omic” data, leading to the discovery of previously unknown networks of interconnected genes and molecular interactions. Nonetheless, almost all these analyses cluster the genes according to their expression levels in large numbers of (usually unmatched) samples (e.g., time series, different tissues, experimental conditions, races/strains, sexes) to reveal system-level properties of prognostic genes (e.g., [6,7,8]). In doing so the authors implicitly assume that gene networking is unique (no alternative wiring), universal (the same regardless of race/strain, age, sex, tissue etc.) and rigid (no remodeling during ageing, disease progression or in response to environmental changes). 

However, when two atoms, nitrogen and oxygen, can combine in several ways (NO, NO_2_, N_2_O, N_2_O_3_, N_2_O_4_, and N_2_O_5_), each combination being dominant in particular conditions, how to conceive that tens of far more complex entities (genes) can form unique, universal and rigid pathways? Therefore, we consider that the genes responsible for a particular biological process (say intercellular Ca^2+^-signaling) can be networked in various ways, depending on the nature of the biological system and can remodel in response to external stimuli. As such, the correlations between expressions of genes are not fixed forever, but dynamic and subject to change to optimize the functional pathways they are involved in. Distinct from the common paradigm, our correlation analysis determines how genes coordinate their expressions not in in different conditions or time-points but in biological replicas of the same condition/time point. The biological replicas (here four dishes of DRG neurons subjected to the same firing pattern and duration) can be considered as the same system subjected to slightly different environment that do not alter significantly the abundances of their transcripts. 

If the covariance of two genes is tightly linked, they are likely part of a common transcriptional network responding to the stimulus, even though expression of these genes may not change with statistical significance [9]. The coordinated variation in abundance of specific transcripts in neurons assessed simultaneously should reflect expression synchrony of the transcriptional networks that are cooperatively engaged in response to a specific stimulus. As such, the coordination analysis is expected to reveal whether the cooperation of the transcriptional networks changes when the neurons are forced to fire and depends on the pattern and duration of the stimulation. In a previous publication [10], we speculated that the expression coordination enforces the “transcriptomic stoichiometry” of the functional gene networks so that the linked genes are expressed in appropriate proportions. Expressions of two genes may be coordinated in four ways: (i) By both increasing in abundance simultaneously; (ii) by both decreasing in abundance, or (iii–iv) by one gene increasing while the other decreases. There is also the possibility of non-coordination in which changes in one gene expression level are not related to changes in the expression of the other gene. An analysis of covariance between all possible pair-wise combinations of genes in the cell would provide the critical information required to identify functionally interconnected gene networks [11] responding to a stimulus, even though the variation in abundance levels of the transcripts may fluctuate below the threshold criterion of being judged significantly different from a mean (or other measure of central tendency) expression level [12]. 

The experiments analyzed in this report were carried out on mouse dorsal root ganglion (DRG) neurons in cell culture. These neurons were chosen because they are not spontaneously active and, critically, they fire single action potentials in response to a brief electrical pulse, they also do not form dendrites or synapses on themselves. These properties of cultured DRG neurons allow precise experimental control of the firing pattern [13,14]. 

A standard analysis of data from these experiments has been published [15]; expression data are available through the NCBI GEO database (www.ncbi.nlm.nih.gov/geo) accession number GSE84872. The results of that analysis showed that hundreds of genes were regulated by the specific pattern of action potential firing. The analyses presented in this study refer to the transcript abundance variability and expression coordination, the other two independent measures in addition to the average expression level that can be associated to every neuronal gene in each stimulation paradigm. These analyses were applied to determine whether and how the control of the transcript abundances changes and functional pathways remodel after electrical stimulation. Similar types of statistical variation and covariance methods have been used by us to ascertain the reconfiguration of gene networks in cancer [16,17], infectious [18] and neurological diseases [19,20,21], to name just a few. 

93 genes related to the calcium signaling (CAS), 934 transcription factors (TRF) and 41 voltage-dependent ion channels were selected using Kyoto Encyclopedia of Genes and Genomes (https://www.kegg.jp) and Gene Ontology (www.geneontology.org). We decided to analyze TRF genes because they modulate the expression of all other genes. CAS genes were analyzed owing to the important role of calcium signaling in controlling almost all major cellular processes. This is especially relevant in neurons, where Ca^2+^ signaling is a critical component linking action potential firing with intracellular signaling pathways and gene expression. Finally, we analyzed the voltage-dependent ion-channels because they are the most sensitive to the electrical activity of the neurons.

Our results show that, in addition to regulating the expression level of numerous genes, the temporal pattern of action potential firing profoundly modulates how genes are networked in functional pathways. Moreover, the transcriptomic landscape of the interaction between transcription factors and calcium signaling genes, and the coordination of the potassium voltage-gated channels with calcium, sodium, and anion voltage dependent channels are strongly influenced by the stimulation pattern and duration. 

## 2. Materials and Methods 

### 2.1. Primary Neuronal Cell Culture

All experiments were conducted in accordance with animal study protocols approved by the NICHD Animal Care and Use Committee (approval code 17-049). For dissociated DRG cell culture, multi-compartment Campenot chambers made of Teflon, were affixed by vacuum grease to collagen coated 35 mm cell culture dishes as previously described [15]. Neurons were dissociated from spinal cords of 13.5-day-old mouse embryos and plated at a density of 0.5 × 10^6^ cells per mL into each side compartment in Eagle’s MEM base media with the addition of a custom N3 supplement, 5% horse serum and 100 ng/mL nerve growth factor. Non-neuronal cell proliferation was inhibited by treatment with 13 μg/mL fluoro-2-deoxyurindine (Sigma, St. Louis, MO, USA) one day following plating for 5 days. Culture conditions have been optimized to enrich for medium-large type DRGs. Genetic markers of small type (nociceptive) neurons, Tyrosine hydroxylase and P2rX3 are expressed at low levels. Cultures were subsequently used for experiments 3–4 weeks after plating by which time axons extend from the side compartments into the central compartment under the central barriers and could be electrically stimulated. These cultures contain no detectable Schwann cells or astrocytes. The Schwann cell specific isoform of the cell adhesion molecule L1 is undetectable by RT-PCR [22], and the astrocyte marker *gfap* is not detected by microarray [15] or RNA sequencing (in press).

### 2.2. Electrical Stimulation of DRG Neurons

DRG cultures with axon outgrowth under the Teflon barrier 23 weeks post plating were selected for electrical stimulation. A complete media change to media lacking NGF and sera was carried out and stimulating lids were placed onto each dish. Cultured cells were then placed back into the incubator and left undisturbed overnight. The following day, cultures were stimulated through platinum electrodes in contact with media on opposite sides of the Teflon barrier and the bulk media. Stimulation parameters and responses to stimulation have been reported previously [15]. For this study, either 18 actions potentials at 10 Hz, repeated every min (18/1) or 90 action potentials repeated every 5 min (90/5) were delivered by a 6V, 0.2 ms biphasic pulse for either 2 h or 5 h. Neurons were stimulated, and total RNA extracted for microarray analysis immediately following stimulation [15]. Results were compared to those obtained from unstimulated neurons.

### 2.3. Microarray Analysis

Microarray experiment was carried out in four replicas of each of the five stimulation conditions on the Agilent-026655 Whole Mouse Genome Microarray 4 × 44K v2 two-color microarray platform GPL10333 as previously described [15]. All control spots, spots affected by local corruption, and spots with foreground fluorescence less than twice the background in any of the 20 samples were disregarded. Data were normalized to the median of valid spots by an iterative method alternating intra- and inter-array normalization until the overall maximum error of estimate became less than 5%. The 39,430 valid spots in each of the 20 samples probed redundantly 13,974 distinct transcripts, forming 97,629,351 distinct pairs. The redundancy was not uniform among the transcripts; although most transcripts were probed by single spots, others were probed by up to 28 spots. By profiling four (or more) biological replicas one gets with acceptable statistical significance, for each expressed gene, three independent measures: (i) average expression level, (ii) expression variability and (iii) expression coordination with every other gene in the same stimulation condition. Let us illustrate the independence by considering the expressions in four biological replicas of three hypothetical genes G1 (0.95, 0.98, 1.01, 1.06), G2 (1.05, 1.01, 1.00, 0.94), G3 (1.50, 1.10, 0.90, 0.50), having the same average level (1.00). G1 and G2 have close coefficients of variation (CV1 ≅ CV2 ≅ 5%) but different from G3 (CV3 ≅ 42%). However, G1 is negatively correlated with G2 (ρ_12_ = −0.985) which at its turn is positively correlated with G3 (ρ_23_ = 0.986). Thus, the present study offers not overlapping but complementary perspectives to the previously published results [15] that relied on the average expression levels of the genes in the electrically stimulated DRG neurons. 

### 2.4. Expression Variability and Coordination 

An important descriptive statistic beyond measures of central tendency is the variance in individual gene expression observed within experimental replicates. The dispersion around mean results from both technical noise (measurement precision and uncontrolled experimental method errors) and biological variability. In addition to the stochastic nature of the chemical reactions involved in transcription [23,24], the expression variability reflects the transcriptomic response to the slight differences in the environmental conditions to which each biological replica was exposed. In order to increase the accuracy of the expression results, the Agilent microarrays probe each transcript by multiple spots, albeit not in uniform numbers across the genome. Thus, when combining the expression results of the four biological replicas, for each transcript *i* probed by spot *k (k = 1*, *…*, *R_i_* = number of spot probing that transcript), one gets the average expression level *μ_ik_* and the standard deviation *σ_ik_*. For the purpose to account for the multiple testing of the same gene and to give more credit to the transcripts probed by larger number of spots, we replaced the traditional coefficient of variation CV = σ/μ by the Relative Expression Variability (REV). REV is defined for each gene in each condition as the mid chi-square interval estimate of the pooled CVs for *4R_i_ -1* degrees of freedom and 5% level of significance:

(1) $$REV_{i}^{\left(condition\right)}=\underset{\mathrm{redundancy}\;\mathrm{correction}}{\underset{︸}{\frac{1}{2}\left(\sqrt{\frac{4R_{i}-1}{\chi^{2}\left(4R_{i}-1;0.975\right)}}+\sqrt{\frac{4R_{i}-1}{\chi^{2}\left(4R_{i}-1;\varepsilon /2\right)}}\right)}}\times \underset{\mathrm{pooled}\;\mathrm{CV}}{\underset{︸}{\sqrt{\frac{1}{R_{i}}\sum_{k=1}^{R_{i}}{\left(\frac{s_{ik}^{\left(condition\right)}}{\mu_{ik}^{\left(condition\right)}}\right)}^{2}}}}$$

With *R_i_* = 1,2, …, 28, the redundancy correction took values from 0.960 (*R_i_* = 28) to 1.566 (*R_i_* = 1). Expression of some genes is more variable than others, as different genes are regulated by distinct mechanisms in an intricate network of regulatory pathways. Immediate early genes, for example, would be expected to exhibit higher variance because these genes respond rapidly to a wide variety of stimuli and physiological changes [25]. However, the average contribution of the technical noise to the expression variability tends to become uniform when large numbers of genes are considered. Therefore, differences in the average expression variability in neurons subjected to different stimulation paradigms indicates that the homeostatic factors constraining gene expression are sensitive to the pattern and duration of electrical stimulation. 

Expression variability is an indicator of the cell priorities in controlling the transcript abundances, with genes critical for the cell survival allowed the least expression variation (lowest REV) across biological replicas [17].

Expression variability further allows calculation of Pearson product-momentum correlation coefficient [26] between (log_2_) expression levels of gene pairs in biological replicas. We apply this analysis only on results from biological replicas to avoid gene pairing being compromised by exposure to different conditions (here different stimulation paradigms). The analysis identifies the genes whose expressions are significantly (*p* < 0.05) positively or negatively coordinated, and of genes whose expressions are significantly (*p* < 0.05) uncorrelated. The statistical significance of the Pearson correlation coefficient for the number of paired (log_2_) expression levels was determined using a two-tail *t*-test for the degrees of freedom df = 4(biological replicas)*R (number of spots probing redundantly each of the correlated transcripts) − 2. For most gene pairs, df = 4 − 2 = 2. If there are two spots probing each gene, then df = 8 − 2 = 6, for 3 spots df = 12 − 2 = 10 and so on. When both genes are probed by a single spot, then for the four pairs of expression levels in the biological replicas the *p* < 0.05 significant correlation applies for |ρ| > 0.95. If both genes are probed by 2 spots each, then *p* < 0.05 significant correlation is for |ρ| > 0.71, and so on, larger the numbers of probing spots, less Pearson coefficient is necessary for the *p* < 0.05 significant correlation. However, not many genes had exactly the same number of probing spots, in which case we had to consider the pooled values with the redundancy groups of spots. https://www.youtube.com/watch?v=Kc3M5x7125A presents a tutorial on of how to determine the Pearson correlation coefficient and its significance in Excel.

As analyzed by Marbach et al. [27], the Pearson correlation is not the strongest method to infer the gene networks. However, given the influence of the technical noise on the gene expression results, Pearson correlation is good enough for our purpose and use of stronger methods will overkill (increased computational accuracy overshadowed by the technology imprecision). Nonetheless, our analysis still yields several (hopefully less than 5% positive and 5% negative) spurious associations, but we believe that, regardless of method, correlating expressions across biological replicas is far more appropriate than correlating expressions in different conditions.

The selected genes formed 4278 distinct CAS–CAS gene pairs, 435,711 TRF–TRF pairs, 86,862 CAS–TRF pairs and 820 pairs of voltage-gated ion channels that were further analyzed for expression correlation and interplay. We have developed a Python version that performs the coordination analysis for the entire transcriptome and then sort the pairs according to their GO category in less than 10 min instead of days in Excel [17].

### 2.5. Genomic Fabric Topology

Pair-wise relevance (PWR) analysis [11,18] was used to determine the topology of the most inter-coordinated and stably expressed transcriptome (termed genomic fabric, [9,28]) associated to a functional pathway in each condition. PWR score encompasses the relative expression levels, the relative controls and the correlation of the two considered genes, *i* and *j*. 

(2) $$\begin{array}{l} PWR_{ij}^{\left(condition\right)}=\frac{\mu_{i}^{\left(condition\right)}\mu_{j}^{\left(condition\right)}}{{\left(\bar{\mu^{\left(condition\right)}}\right)}^{2}}\times {\left(\rho_{ij}^{\left(condition\right)}\right)}^{2}\times \frac{{\left(\bar{REV^{\left(condition\right)}}\right)}^{2}}{REV_{i}^{\left(condition\right)}REV_{j}^{\left(condition\right)}}\;,\;where:\\ \bar{\mu^{\left(condition\right)}}\;=\;\frac{1}{N}\sum_{k=1}^{N}\mu_{k}^{\left(condition\right)}\;,\;N=\mathrm{number}\;\mathrm{of}\;\mathrm{unigenes}\;,\;\bar{REV^{\left(condition\right)}}\;=\;\frac{1}{N}\sum_{k=1}^{N}REV_{k}^{\left(condition\right)}\\ \rho_{ij}^{\left(condition\right)}=\;\mathrm{Pearson}\;\mathrm{correlation}\;\mathrm{beween}\;\mathrm{the}\;\mathrm{expression}\;\mathrm{levels}\;\mathrm{of}\;\mathrm{genes}\;i\;\mathrm{and}\;j \end{array}$$

PWR analysis was also used to determine the interplay between the calcium signaling and the transcription factors (in Equation (2), *i* is a CAS gene and *j* is a TRF). Because both the correlation analysis and the PWR analysis are symmetrical to the switch of the genes within the pair, neither one can determine what gene in the pair is the regulator and what gene is the regulated.

## 3. Results

### 3.1. Relative Expression Variability

**Figure 1:**
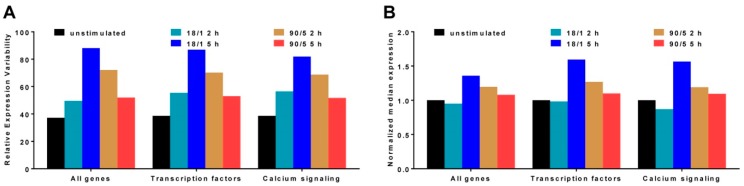
Average relative expression variability (REV) (**A**) and normalized median expression (**B**) of all quantified (ALL, 13,974 distinct genes), calcium signaling related genes (CAS, 93 genes) and transcription factors (TRF, 934 genes) after electrical stimulation at two different patterns for 2 and 5 h. The means of the REV distributions in stimulated conditions were significantly different from the control (unstimulated) ones as measured by the *p*-values of the heteroscedastic (two-sample unequal variance) *t*-test of the two means equality: 18/1 2 h (ALL < 10 ^−308^; CAS 6.28 × 10 ^−6^; TRF 3.78 × 10 ^−51^), 18/1 5 h (ALL 3.2 × 10 ^−216^; CAS 2.76 × 10 ^−11^; TRF 2.14 × 10 ^−132^), 90/5 2 h (ALL <10 ^−308^; CAS 3.59 × 10 ^−9^; TRF 2.557 × 10 ^−97^), 90/5 5 h (ALL <10 ^−308^; CAS 7.10 × 10 ^−6^; TRF 1.52 × 10 ^−37^).

The REV values are reported in Supplementary Table S1 for the 93 adequately quantified genes that are known to be associated with calcium signaling, (CAS) and Supplementary Table S2 for the 934 transcription factors (TRF). Figure 1A presents the average REV values for all quantified genes (ALL) and for the TRF and CAS gene groups. For comparison, Figure 1B presents the median expression level of the same groups of genes normalized to the level of unstimulated neurons. Our data show that not only the expression level (as discussed in the previous publication, [15]) but also the average variation is sensitive to the electrical stimulus, and the pattern and duration of stimulation. Unstimulated neurons showed the smallest expression variation of genes, and genes in neurons subjected to 18 action potential bursts at 10 Hz, repeated every minute for 5 h exhibited the largest expression variation for all these three groups of genes considered. 

Table 1 presents the two most stably expressed (lowest REVs) and the two most unstably expressed (highest REVs) calcium signaling genes and transcription factors in all stimulation paradigms. For the same genes, Table 1 presents also the average expression levels in all conditions, indicating the significant regulation. REV differences across the conditions indicate changes in the neurons’ priorities in controlling the transcript abundance. For instance, the abundance of *Itpkc*, the most stably expressed CAS gene in the unstimulated neurons, associated with NGF-driven neurite outgrowth [29], becomes much less controlled in stimulated neurons. The differences are even more spectacular for the purinergic receptor *P2rx4* which is the most stably expressed at 18/1 2 h but becomes very variably expressed if the stimulation is extended for 5 h (and also at 90/5 2 h). The case of *P2rx4* indicates that the neuronal homeostatic mechanisms are highly dynamic and changes not only with the pattern but also with the duration of the stimulation. 

### 3.2. Expression Coordination

**Figure 2:**
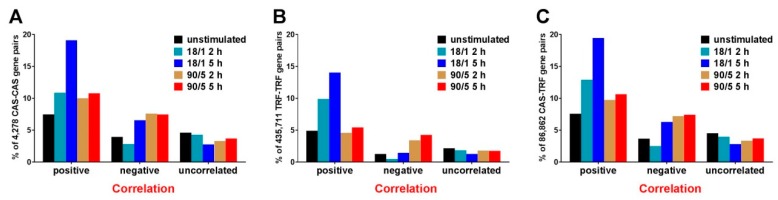
Pearson correlation of gene expressions. Percentage of (*p* < 0.05) significantly uncorrelated and positively or negatively correlated (**A**) calcium signaling (CAS–CAS) gene pairs, (**B**) transcription factor (TRF–TRF) gene pairs and (**C**) CAS–TRF gene pairs in all experimental conditions. Note that the percentage of positive coordination is substantially higher for the 18/1 stimulation pattern at 5 h and strong positive coordination of the calcium signaling genes with the transcription factors. The difference between 100% and sum of the represented percentages is composed by the gene-pairs whose coordination did not meet the statistical evidence to be categorized as significantly uncorrelated, or as significantly positively or negatively correlated.

The level of expression coordination was determined for all 97,629,351 distinct pairs of transcripts under each experimental condition. A summary of the 4728 CAS–CAS pairs, 435,711 TRF–TRF pairs and 86,862 CAS–TRF pairs are shown in Figure 2A–C respectively. The (*p* < 0.05) significant coordinations of the gene pairs were categorized as positive, negative or independent across all five conditions. Figure 2 shows the substantial dependence of the expression coordination on the stimulation pattern, while the extended duration (5 h vs. 2 h) was influential only for the 18/1 pattern. Interestingly, the majority of CAS and TRF genes were positively coordinated not only within their respective fabrics (CAS–CAS, TRF–TRF) but also in the CAS–TRF interplay, indicating synchronous co-regulation. 

### 3.3. Expression Level and Correlation of the Voltage-Gated Ion Channels Depends on Electrical Stimulation 

**Figure 3:**
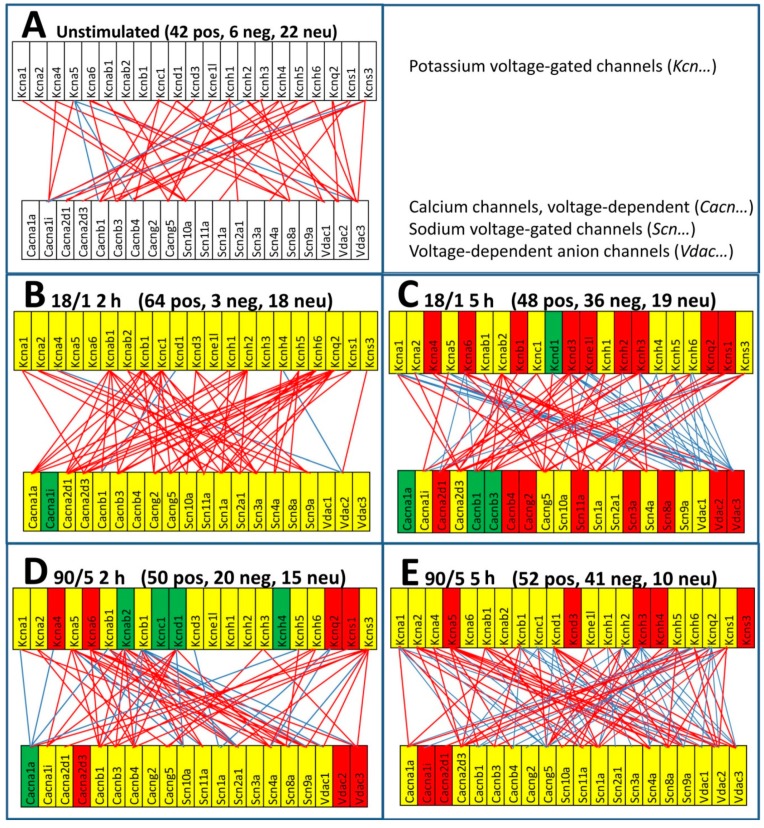
Expression regulation and coordination of some voltage-gated channels in stimulated DRG neurons. (**A**) unstimulated neurons. (**B**) neurons stimulated for 2 h with 18/1 pattern. (**C**) neurons stimulated for 5 h with 18/1 pattern. (**D**) neurons stimulated for 2 h with 90/5 pattern. (**E**) neurons stimulated for 5 h with 90/5 pattern. Red/green background of the gene symbol indicates significant up-/down-regulation of that gene in that stimulation condition with respect to unstimulated neurons. Yellow background indicates that the expression change was not statistically significant. Red/blue line indicates that the expressions of the linked genes are significantly positively/negatively correlated. Note the substantial differences among the stimulation paradigms. Missing lines indicate that the expression coordination between the corresponding genes were not statistically significant.

Voltage-gated ion channels are transmembrane ion transporting proteins that are activated in response to changes in the membrane potential. We analyzed whether the action potential firing has consequences on the expression level and coordination of the genes encoding such channels. Figure 3 presents a part of this analysis depicting how the expression levels and correlation of certain voltage-gated ion channels change with the stimulation paradigm. 

It is interesting to compare the results from Figure 3 with those obtained on DRG neurons stimulated with 10 Hz pulses lasting for 0.5 sec every 8 sec, 12 h per day, for 5 days [30]. With respect to non-stimulated neurons, that much longer stimulation increased neuronal firing frequency and activation, did not affect significantly *Scn3a* (Nav1.3) but significantly down-regulated *Scn10a* (Nav1.8) and *Scn11a* (Nav1.9), and altered calcium currents as previously shown [30]. Under the current stimulation paradigms, we found *Scn3a* as unaltered in all conditions but 18/1 5 h, where it was significantly up-regulated by 2.06x. We found also that *Scn10a* was not affected by any stimulation, while *Scn11a* was up-regulated only in 18/1 5 h (by 2.10x). The differences between the new results and the 2003 results illustrate again the sensitivity of the transcriptome to the environmental conditions (here different patterns of stimulation). However, the coordination analysis revealed previously unknown coupling between these sodium channels and the potassium channels. Interestingly, the positive correlations of *Scn3a* with *Kcna2, Kcnb1, Kcnc1,* and *Kcnd3* in 18/1 2 h neurons are not present in unstimulated neurons and are canceled in all other stimulation paradigms, indicating how responsive are the gene networks to the neuronal electrical activity. However, *Scn3a* establishes a new negative correlation at 90/5 5 h with the pseudogene *Kcne1l* (Potassium voltage-gated channel, Isk-related family, member 1-like, pseudogene), known to slow the activation of the Kcnq1 channel [31].

### 3.4. Coordination Networks

**Figure 4:**
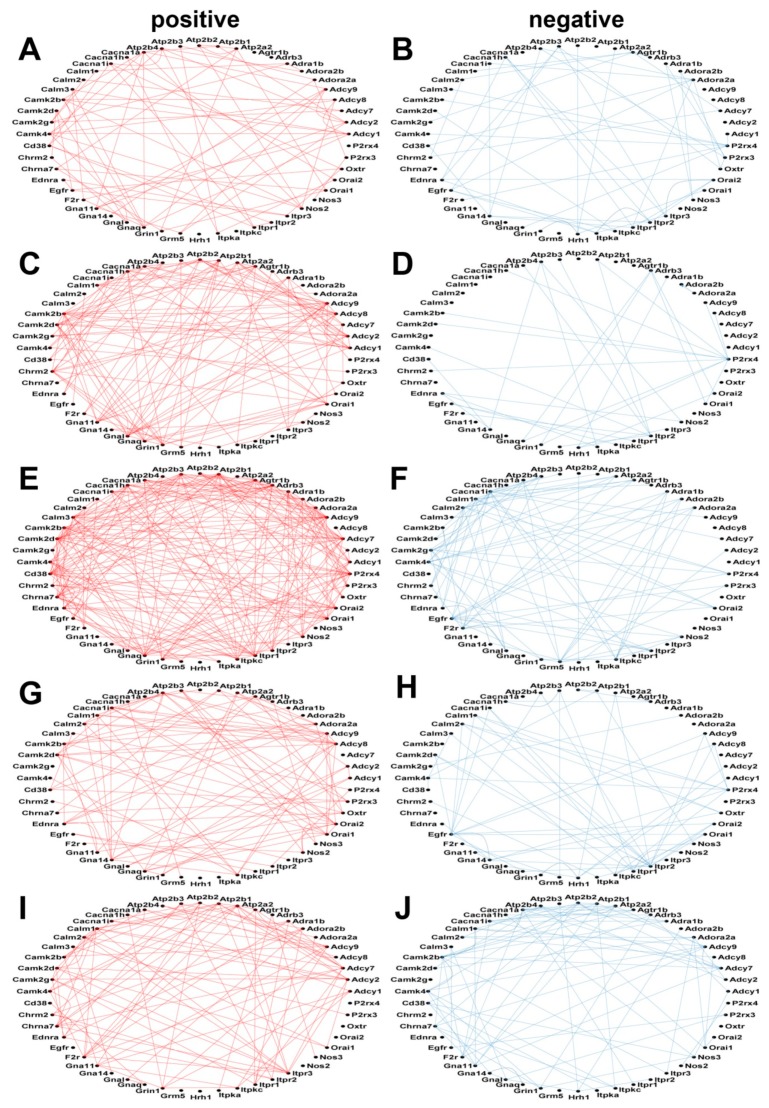
Positive and negative expression coordination of the first 50 alphabetically ordered calcium signaling genes in each pattern of stimulation. (**A**) positive correlations in unstimulated neurons. (**B**) negative correlations in unstimulated neurons. (**C**) positive correlations in neurons stimulated for 2 h with 18/1 pattern. (**D**) negative correlations in neurons stimulated for 2 h with 18/1 pattern. (**E**) positive correlations in neurons stimulated for 5 h with 18/1 pattern. (**F)** negative correlations in neurons stimulated for 5 h with 18/1 pattern. (**G**) positive correlations in neurons stimulated for 2 h with 90/5 pattern. (**H**) negative correlations in neurons stimulated for 2 h with 90/5 pattern. (**I**) positive correlations in neurons stimulated for 5 h with 90/5 pattern. (**J**) negative correlations in neurons stimulated for 5 h with 90/5 pattern. A red/blue line indicates a statistically significant (*p*-value < 0.05) positive/negative coordination of the linked genes. Missing lines indicate that the expression coordination between the corresponding genes is not statistically significant.

**Figure 5:**
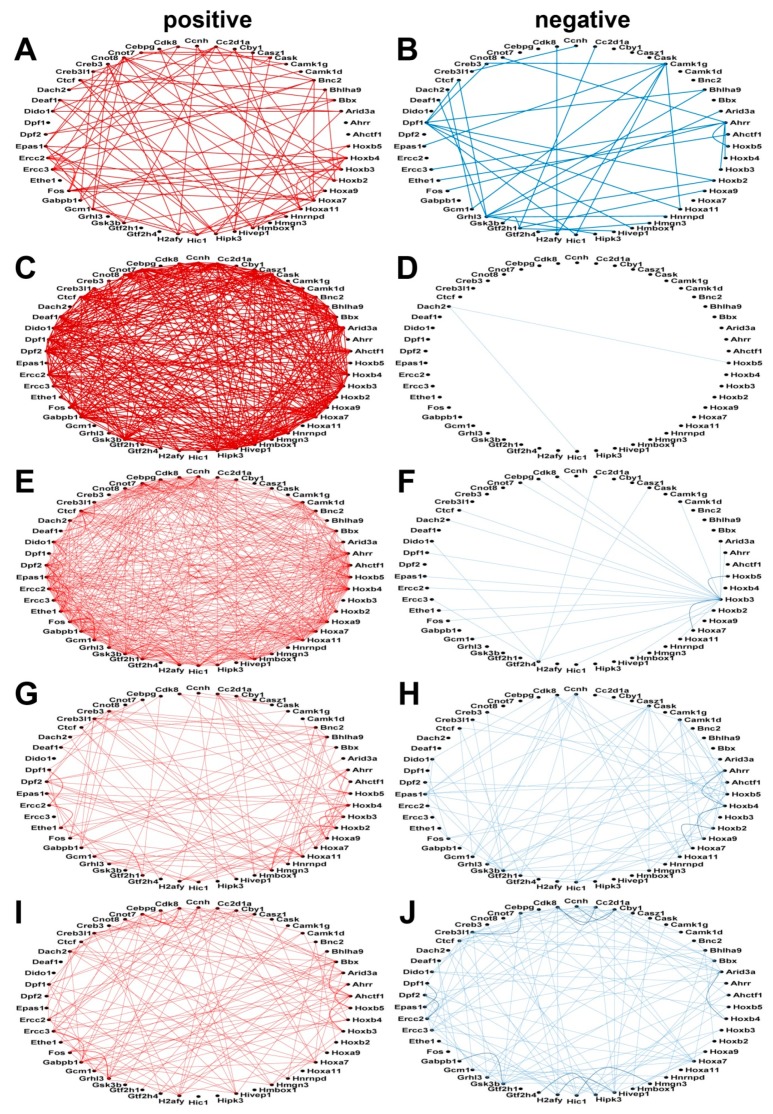
Positive and negative expression coordination of 50 alphabetically ordered, randomly selected transcription factor genes (TRF) in all experimental conditions. (**A**) positive correlations in unstimulated neurons. (**B**) negative correlations in unstimulated neurons. (**C**) positive correlations in neurons stimulated for 2 h with 18/1 pattern. (**D**) negative correlations in neurons stimulated for 2 h with 18/1 pattern. (**E**) positive correlations in neurons stimulated for 5 h with 18/1 pattern. (**F**) negative correlations in neurons stimulated for 5 h with 18/1 pattern. (**G**) positive correlations in neurons stimulated for 2 h with 90/5 pattern. (**H**) negative correlations in neurons stimulated for 2 h with 90/5 pattern. (**I**) positive correlations in neurons stimulated for 5 h with 90/5 pattern. (**J**) negative correlations in neurons stimulated for 5 h with 90/5 pattern. A red/blue line indicates a statistically significant (*p*-value < 0.05) positive/negative coordination of the linked genes. Missing lines indicate that the expression coordination is not statistically significant. As in the case of CAS genes, the TRF network responds to the action potential firing pattern.

**Figure A1:**
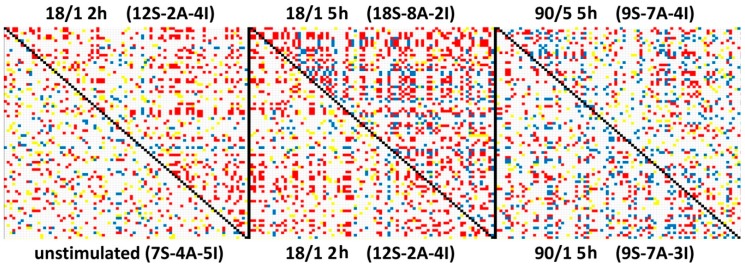
Comparison of the expression coordination of the 93 adequately quantified calcium signaling genes among stimulation paradigms. A red/blue/yellow square indicates a statistically significant (*p* value < 0.05) positive/negative/independent coordination while a blank square indicates lack of statistical significance to assess the type of coordination. Numbers indicate the percentages of gene pairs exhibiting statistically significant positive (S), negative (A) and independent (I) coordination in the respective pattern of stimulation.

Figure 4 and Figure 5 illustrate how the CAS and the TRF genes are coordinately expressed among themselves in resting state neurons (unstimulated) or after stimulation with the 18/1 or 90/5 pattern of action potential firing for 2 h or 5 h. Networks of subsets of only 50 CAS and TRF genes are presented for the sole purpose of simplifying the graphical display. (A full comparison of the expression coordination of calcium signaling genes in all stimulation paradigms is provided in Appendix A, Figure A1). The number of positively correlated pairs increased sharply 2 h after electrical stimulation by the 18/1 pattern and increased even further by 5 h. 

**Figure A2:**
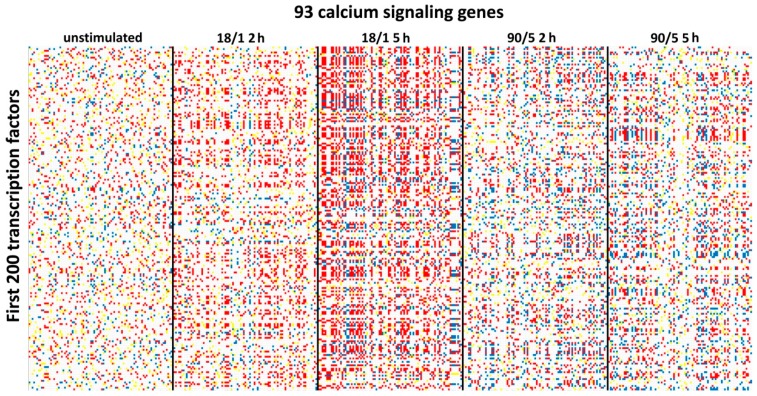
Part of the expression coordination of the all 93 quantified calcium signaling genes with 200 randomly selected (out of 934) transcription factors. A red/blue/ square indicates a statistically significant (*p* value < 0.05) positive/negative coordination, an yellow square indicates no correlation, while a blank square indicates lack of statistical significance to assess the type of correlation. Note the significantly higher degree of coordination between the transcription factors and the calcium signaling genes for the 5 h stimulation with the 18/1 pattern.

The similarity in responses in coordinated expression between pairs of calcium related genes and pairs of transcription factor genes, suggests interaction between these two types of genes in a transcriptional network responding to patterned action potential firing. Figure A2 from Appendix A presents the coordination network of the 93 CAS genes with the 200 randomly selected TRFs.

### 3.5. Gene Pairing Can Be Reversed by Changing Pattern or Duration of Stimulation 

Interestingly, in addition to considerably increasing the percentage of significantly coordinately expressed gene pairs, the electrical stimulation also reversed the type of coordination existing in the non-stimulated condition. Coordination reversal may occur not only in stimulated versus unstimulated neurons but also when changing the pattern of stimulation for the same duration or the duration for the same pattern. For instance, Table 2 presents all significantly coordinated CAS–CAS gene pairs by 90/5 for 2 h whose coordination is opposite to that for the 5 h duration (e.g., *Adcy8-Calm2)* or in the pattern 18/1 for the same 2 h duration (e.g., *Atpb3-Grm5*) or in the unstimulated neurons (e.g., *Adora2a-Cd38*). Table 2 illustrates also the significantly coordinated TRF–TRF pairs in neurons stimulated with the 18/1 pattern for 2 h, whose coordination is opposite to that for the same stimulus but for 5 h duration (e.g., *Camk1d-Hoxb3*) or in the pattern 90/5 for the same 2 h duration (e.g., *Bnc2-Gsk3b*). Together these results indicate that not only the pattern in which the same number of action potentials are delivered but also the duration of delivery profoundly alters the coordinated activity between transcription factor genes. 

### 3.6. Interplay of Calcium Signaling Genes and Transcription Factors

**Figure 6:**
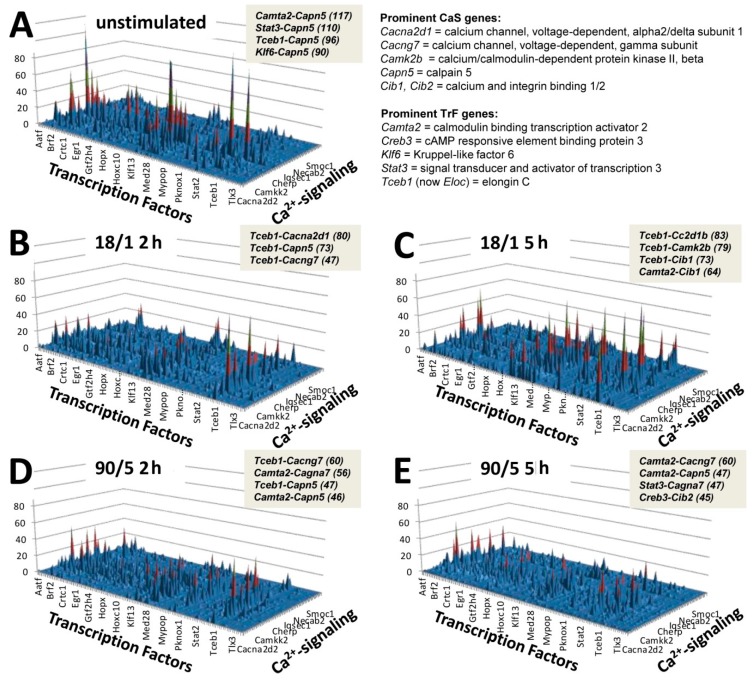
Pair-wise relevance (PWR) analysis of the interaction between 50 calcium signaling genes (CAS) and 50 transcription factors (TRF) in all patterns of stimulation. (**A**) unstimulated neurons. (**B**) neurons stimulated for 2 h with 18/1 pattern. (**C**) neurons stimulated for 5 h with 18/1 pattern. (**D**) neurons stimulated for 2 h with 90/5 pattern. (**E**) neurons stimulated for 5 h with 90/5 pattern. The medallions present the relevant TRF–CAS gene pairs (and their PWR scores) in each condition.

Figure 2C and Figure A2 from Appendix A clearly indicate interaction between CAS and TRF genes in a transcriptional network responding to patterned action potential firing. To examine this quantitatively, all possible 86,862 pairwise combinations of 93 calcium signaling related genes and 934 transcription factors were scored by the pair-wise relevance index (PWR). The analysis produced for each stimulation paradigm a 3D plot partially represented in Figure 6. 

In Figure 6, higher peaks represent the most prominent CAS–TRF pairs of coordinately expressed genes. The hypothesis is that the interwoven coordinated activity between pairs of genes in these two categories will be altered by specific patterns of action potential. A CAS–TRF gene-pair becomes prominent for the neural transcriptome when the expressions of the two genes are not only high but also when they are strongly coordinated, while under strict control by the homeostatic cell mechanisms. 

## 4. Discussion

Changes in gene expression in DRG neurons after action potential stimulation were analyzed in terms of the variability and coordinated activity between pairs of calcium signaling related genes and transcription factor genes. The analysis provides an important and different perspective on the activity-dependent transcriptional response in neurons than is obtained by simple identification of genes that are up or down regulated significantly following stimulation. Not only the expression levels of the neuronal genes are affected by stimulation but also the organizational principles of the (here neuronal) transcriptome [32] and the homeostatic mechanisms controlling the transcript abundances. Importantly, the coordinated activity of gene pairs is markedly changed by the pattern and duration of action potential firing. 

The specific functional consequences of the two (18/1 and 90/5) patterns of action potential firing are not known; indeed, it is uncertain whether any persistent functional consequences would be produced by action potential firing in these two patterns. Determining the possible functional consequences of firing neurons in these two patterns for 2 or 5 h was not the objective of the present series of experiments. Instead, these experiments test whether the transcriptional network is altered by differences in the pattern of action potential firing detected by analyses of expression variability and expression coordination between pairs of genes, even if the levels of expression of these genes may not change sufficiently at the *p* < 0.05 level. However, the specific pairs of transcription factors and calcium signaling genes that are coordinately activated and with high prominence that are revealed here will provide a valuable database for researchers in studies related to a wide range of functions.

By hypothesis, we expected increased correlated activity between pairs of genes after action potential stimulation, and this was observed. However, we had no a priori expectation that the majority of gene pairs would be positively correlated; that is, both genes increasing or both decreasing in expression level in tandem after stimulation. A surprising result was that so few genes were negatively correlated (one gene increased while the other decreased in expression). Since a gene product in a network may have either a facilitatory or inhibitory influence, negatively correlated gene pairs could either promote or inhibit a pathway or functional response, depending on whether the inverse correlation uncouples two genes having the same direction of influence or disinhibits opposing influences. The finding of so few negatively correlated gene pairs, and an increase in the number of negatively correlated gene pairs with more prolonged impulse trains and prolonged stimulation, indicates that calcium-regulated genes and transcription factor genes in these neurons tend to influence the transcriptional network in the same direction in response to action potential firing. 

Pair-wise relevance analysis illustrated in Figure 6 show many interesting gene pairs in stimulated and unstimulated cells. Unstimulated cells show pairs of transcription factors and calcium genes which are primarily regulators of metabolic cellular processes, for example cholesterol metabolism, such as the transcription factor *Camta2* and the calcium signaling gene *Capn5*, both of which are upregulated in cells in which cholesterol metabolism is altered [33]. Three of the four top pairings are unique to the unstimulated condition, *Tceb1-Capn5* is the only pairing carried over into any of the other conditions, 18/1 2 h and 90/5 2 h and the numbers are lower in both conditions. 

The pairings in the 18/1 pattern at 2 h and 5 h stimulation time are dominated by the transcription factor *Tceb1*, however the calcium signaling genes in each pairing are unique to each time point. This likely shows a change in transcriptional priorities and calcium signaling in the simulated cells from cellular metabolic activity signature seen in the unstimulated condition to a more focused genetic signature related to the decoding of external signals. In support of this hypothesis it has recently been demonstrated that the activity of transcription factor *Tceb1* is regulated by experience dependent neuronal plasticity driven by specific external stimuli in *Caenorhabditis elegans* [34]. *Tceb1* is a transcriptional regulator of transcription elongation, therefore it has the capacity to be a common regulator of many genes, including calcium signaling genes, which are controlled by neuronal activity firing patterns. Multiple steps in the transcriptional process are dependent upon calcium signaling, including transcriptional elongation and termination [35]. After 5 h of the 18/1 stimulus pattern a different group of calcium signaling genes are paired with *Tceb1* and a novel pairing of *Camta2-Cib1* is seen. As is observed at 2 h, the calcium signaling genes have varied roles in intracellular signaling and are all linked to neuronal function. 

The gene pairings with the 90/5 stimulus pattern show a different set of predominating transcription factors and novel gene pairings of transcription factors and calcium genes from the 18/1 stimulus and unstimulated cells, particularly after 5 h of stimulus. After 2 h of stimulus we again see the *Tceb1-Cacng7* pairing, as we do with the 18/1 2 h time point, however a unique pairing of *Camta2-Cagna7*, not seen with the 18/1 stimulus is observed, indicating perhaps a regulated gene network specific to the 90/5 stimulus pattern. However, after 5 h of 90/5 stimulus we observe not only unique gene parings but also a new set of transcription factors and calcium genes. *Creb3* transcription factor is a regulator of cytosolic calcium concentration [36] and memory maintenance in *Aplysia* [37] and is paired with *Cib2*, a gene required for calcium dependent mechanotransduction and growth of auditory hair cells [38]. Therefore, many of the novel gene pairs identified in the PWR analysis have direct relevance to synaptic plasticity and activity-dependent gene expression in the nervous system. 

The gene pairs in the PWR analysis are computationally related, but may not influence each other directly if they operate in different pathways. However, with or without direct interaction, all functional pathways should work in a coordinated manner to optimize the cellular processes. In this respect, the covariance analysis provides a unique screen for possible biological interactions which could not be found by the traditional method of comparing expression levels of genes.

The emphasis here has been on presumed activity-dependent effects on transcription on calcium signaling related genes and transcription factor genes. Nonetheless, action potential firing activates multiple intracellular signaling networks, notably via intracellular calcium signaling and subsequent activation of kinases and phosphatases, that impinges on the full spectrum of biological processes determining the abundance of specific gene transcripts, from gene transcription to mRNA degradation. The level of expression of any gene transcript measured by microarray in these experiments reflects the net result of all the multiple cellular processes influencing mRNA abundance. Although this analysis focused on calcium signaling related genes and transcription factors, in principle, the findings should apply to other gene pairs and to other cellular processes impacted by action potential firing that influence multiple aspects of gene expression.

It is well-known that the correlation metric determines how genes are clustered. However, we do not construct gene regulatory networks according to their responses across conditions (including knockouts, as algorithms such as ENET [39], GENIE3, and BTNET [40] do) but coordination networks to maintain the “transcriptomic stoichiometry” in response to slight environmental fluctuations as seen in biological replicas. Transcriptomic stoichiometry is an extension of Proust’s Law of Definite Proportions and Dalton’s Law of Multiple Proportions from chemistry, stating that the optimal functional pathways require the composing genes being expressed in adequate proportions. 

Decades-long debate did not yet decide whether the *p*-value should be corrected for multiple testing to establish whether a gene is significantly regulated when comparing two conditions. The correction, using either Benjamini and Hochberg [41], Benjamini and Yekutieli [42], Bonferroni [43], Hochberg and Benjamini [44], Holm [45], or Hommel [46] method, is intended to reduce the number of false positives. However, such correction may also eliminate a good number of true positives. Moreover, it is disputable that profiling the expressions of two distinct genes (say a sodium channel and a transcription factor) is a double testing since different transcripts are hybridized on different spotted sequences, so there are different sets of H-bonds to be formed. Therefore, consistent with our long-standing protocol, we apply a Bonferroni-like correction only for groups of spots probing redundantly the same transcript.

## 5. Conclusions

The study has some limitations owing to the inherent technical noise of the microarray platform. However, the strikingly different patterns of coordinated gene expression seen in the five different stimulus conditions are consistent with expected differences in calcium signaling produced by the two action potential firing patterns and with the apparent homeostatic changes over stimulus time. Overall, these results indicate that the transcriptome is being altered in a coordinated manner, despite signal-to-noise considerations. 

## Figures and Tables

**Table 1:** Relative expression variability (REV) scores and average expression levels for the most stably (low REV, blue background) and unstably (high REV, purple background) expressed calcium signaling genes and transcription factors in each stimulation paradigm.

Gene	Description	Rev					Average Expression Level				
		UNST	18/1 2 h	18/1 5 h	90/5 2 h	90/5 5 h	UNST	18/1 2 h	18/1 5 h	90/5 2 h	90/5 5 h
Itpkc	Inositol 1,4,5-trisphosphate 3-kinase C	4.08	69.86	23.45	46.48	21.24	0.85	0.78	0.60	0.78	0.88
Phka1	Phosphorylase kinase alpha 1	5.09	28.95	74.08	58.14	37.93	1.63	1.47	3.55	1.97	2.01
P2rx4	Purinergic receptor P2X, ligand-gated ion channel 4	16.37	3.95	112.25	117.08	19.17	9.29	10.71	26.82	25.88	12.50
Camk2g	Calcium/calmodulin-dependent protein kinase II gamma	34.96	9.81	22.38	15.84	45.57	10.06	7.05	8.58	8.90	8.11
Itpr1	Inositol 1,4,5-trisphosphate receptor 1	40.17	55.94	10.70	77.73	71.53	0.42	0.41	1.31	0.54	0.46
Nos3	Nitric oxide synthase 3, endothelial cell	16.83	43.24	15.12	112.12	51.14	0.44	0.46	0.39	0.38	0.43
Ptger1	Prostaglandin E receptor 1 (subtype EP1)	43.58	36.53	49.36	13.32	30.79	0.50	0.35	0.60	0.42	0.47
P2rx3	Purinergic receptor P2X, ligand-gated ion channel, 3	35.66	32.39	37.78	14.48	44.31	3.43	3.04	2.77	4.01	3.69
Prkacb	Protein kinase, cAMP dependent, catalytic, beta	18.42	65.01	79.03	32.26	14.03	2.90	2.37	4.47	2.69	2.66
Tnnc2	Troponin C2, fast	38.88	16.41	104.57	65.73	14.63	0.15	0.18	0.38	0.16	0.18
Gna14	Guanine nucleotide binding protein, alpha 14	111.31	108.18	65.77	91.80	135.19	2.38	2.89	4.46	2.43	2.29
Itpka	Inositol 1,4,5-trisphosphate 3-kinase A	135.85	70.23	43.64	52.15	50.27	0.31	0.27	0.31	0.36	0.48
Adcy8	Adenylate cyclase 8	53.29	140.51	129.81	161.86	49.72	0.31	0.28	0.19	0.33	0.30
Adcy1	Adenylate cyclase 1	72.76	187.74	83.73	96.58	49.77	1.18	0.93	2.03	1.66	1.20
Cacna1i	Calcium channel, voltage-dependent, alpha 1I subunit	15.41	13.73	241.17	111.72	41.66	0.17	0.15	2.12	0.14	0.28
P2rx6	Purinergic receptor P2X, ligand-gated ion channel, 6	43.00	81.81	247.15	69.38	78.50	0.18	0.19	4.19	0.21	0.22
Atp2b4	ATPase, Ca++ transporting, plasma membrane 4	32.40	27.58	64.75	185.86	19.62	0.22	0.20	0.19	0.38	0.27
Pdgfrb	Platelet derived growth factor receptor, beta polypeptide	89.66	59.63	71.95	189.03	53.23	1.11	1.09	3.97	4.29	1.19
Phkb	Phosphorylase kinase beta	18.64	59.47	91.03	46.67	114.62	0.53	0.46	1.01	0.70	0.64
Gna14	Guanine nucleotide binding protein, alpha 14	111.31	108.18	65.77	91.80	135.19	2.38	2.89	4.46	2.43	2.29
Psmd4	Proteasome (prosome, macropain) 26S subunit, non-ATPase, 4	3.58	41.88	22.38	27.56	19.04	7.99	8.28	8.58	9.88	8.81
Meis3	Meis homeobox 3	4.79	44.56	105.78	82.12	42.64	4.43	4.09	9.58	8.58	5.77
Hes2	Hairy and enhancer of split 2 (Drosophila)	60.24	3.20	118.34	92.56	22.53	0.14	0.13	0.95	0.45	0.15
Dmap1	DNA methyltransferase 1-associated protein 1	8.75	3.36	112.71	56.49	32.57	7.51	7.67	5.26	7.70	9.23
Zscan26	Zinc finger and SCAN domain containing 26	37.80	59.71	4.11	57.66	71.56	2.73	2.57	5.16	2.36	2.18
Zfp454	Zinc finger protein 454	71.95	35.51	6.34	75.85	56.53	0.30	0.26	0.74	0.76	0.26
Zfpm1	Zinc finger protein, multitype 1	57.82	27.68	31.25	3.00	32.40	1.98	1.44	1.54	1.28	1.88
Ebf1	Early B cell factor 1	21.01	50.60	41.41	10.42	45.89	5.05	4.48	12.31	7.42	4.10
Lmx1b	LIM homeobox transcription factor 1 beta	44.43	29.51	130.55	137.91	1.37	0.23	0.22	0.92	0.44	0.33
Irf3	Interferon regulatory factor 3	13.04	21.88	55.66	47.89	4.79	5.91	6.49	6.11	6.77	7.51
Fos	FBJ osteosarcoma oncogene	234.09	37.80	36.62	42.38	41.18	0.98	2.70	5.22	4.63	6.20
Hoxa11	Homeobox A11	282.22	274.19	129.55	170.19	222.34	4.82	4.52	6.85	6.61	5.55
Kit	Kit oncogene	139.43	197.96	177.49	152.75	48.95	0.29	0.33	1.02	0.38	0.25
Hoxa11	Homeobox A11	282.22	274.19	129.55	170.19	222.34	4.82	4.52	6.85	6.61	5.55
Esrrb	Estrogen related receptor, beta	46.14	38.10	237.35	206.64	20.94	0.27	0.22	2.57	1.21	0.23
Id3	Inhibitor of DNA binding 3	90.45	77.58	244.49	144.85	85.30	1.05	1.23	2.20	2.13	1.06
Sall4	Sal-like 4 (Drosophila)	30.32	16.78	72.94	186.67	18.01	1.12	1.15	73.32	36.72	1.14
Esrrb	Estrogen related receptor, beta	46.14	38.10	237.35	206.64	20.94	0.27	0.22	2.57	1.21	0.23
Ski	Ski sarcoma viral oncogene homolog (avian)	25.40	82.12	69.90	92.66	211.33	0.31	0.24	0.17	0.26	1.69
Hoxa11	Homeobox A11	282.22	274.19	129.55	170.19	222.34	4.82	4.52	6.85	6.61	5.55

Lower REV indicates stronger control of transcript abundance by the homeostatic mechanisms, while higher REV indicates relaxed control. As indicated in Methods, expression levels were normalized to the median expression of all quantified genes in that condition. Red/green background indicates significant up-/down-regulation with respect to unstimulated (UNST). Note that REV is not related to the average expression level although both gene expression characteristics change with stimulation paradigm.

**Table 2:** Gene expression coordination may be reversed by changing pattern or duration of the electrical stimulation.

Calcium Signaling Genes				
Gene Pair	90/5 2 h	Unstimulated	18/1 2 h	90/5 5 h
Adcy8-Calm2	0.93590			−0.90817
Adcy9-Calm1	0.95850			−0.95630
Adora2a-Cd38	0.93826	−0.95238		
Adora2a-Oxtr	0.93992	−0.98837		
Adra1b-Camk2b	0.93396			−0.92631
Agtr1b-Camk2b	0.98624			−0.90249
Atp2b1-Camk4	−0.90327			0.96986
Atp2b3-Grm5	−0.90199		0.99263	
Atp2b4-Calm2	0.91756			−0.94692
Atp2b4-Gna14	0.98172	−0.94881	−0.94881	
Cacna1i-Gna14	0.95922	−0.98677		
Cacna1i-Itpr1	−0.94637	0.96679		
Calm2-Egfr	−0.90024		0.99080	
Camk2d-Egfr	−0.99649	0.92581		
Camk4-Itpkc	0.92652			−0.90723
Egfr-Itpr1	0.90311		−0.91124	
F2r-Grm5	−0.90302		0.98162	
Transcription Factors				
Gene Pair	18/1 2 h	UNSTIMULATED	18/1 5 h	90/5 2 h
Ahctf1-Grhl3	0.97253			−0.90926
Arid3a-Ctcf	0.90668			−0.95895
Arid3a-Ctcf	0.91114			−0.94827
Bhlha9-Gsk3b	0.93771			−0.98955
Bnc2-Gsk3b	0.96196			−0.98325
Camk1d-Cc2d1a	0.92630			−0.97453
Camk1d-Hoxa7	0.94662			−0.99943
Camk1d-Hoxa9	0.97426			−0.95027
Camk1d-Hoxb3	0.97329		−0.95391	−0.99620
Cask-Hmgn3	0.93393			−0.92576
Ccnh-Gabpb1	0.97091			−0.90340
Cdk8-Hoxb2	0.98575			−0.94405
Cnot7-Hoxb3	0.98709		−0.93779	
Ctcf-Hoxb3	0.99212		−0.91724	
Dach2-Hoxb5	−0.97088		0.96951	
Dpf1-Hmbox1	0.97469	−0.90904		
Dpf1-Hoxb2	0.90242	−0.92939		
Dpf2-Gsk3b	0.95509			−0.99496
Ercc2-Hnrnpd	0.99951			−0.95748
Ercc2-Hoxb3	0.91956		−0.98387	
Fos-Gsk3b	0.95949			−0.91342
Fos-Hoxb3	0.91623		−0.93715	
Gsk3b-Hmgn3	0.91755			−0.99482
Hmbox1-Hoxb3	0.96990		−0.94583	
Hoxa9-Hoxb4	0.94215			−0.91487

Red/blue background indicates significant positive/negative Pearson correlation coefficient. CAS genes: *Adcy8/9* (Adenylate cyclase 8/9), *Adora2a* (Adenosine A2a receptor), *Adra1b* (Adrenergic receptor, alpha 1b), *Agtr1b* (Angiotensin II receptor, type 1b), *Atp2b1/3/4* (ATPase, Ca^++^ transporting, plasma membrane 1/3/4), *Cacna1i* (Calcium channel, voltage-dependent, P/Q type, alpha 1I subunit), *Calm1/2* (Calmodulin 1/2), *Camk2b/d*, *Camk4* (Calcium/calmodulin-dependent protein kinase II beta /delta, IV), *Cd38* (CD38 antigen), *Egfr* (Epidermal growth factor receptor), *F2r* (Coagulation factor II (thrombin) receptor), *Gna14* (Guanine nucleotide binding protein, alpha 14), *Grm5* (Glutamate receptor, metabotropic 5), *Itpkc* (Inositol 1,4,5-trisphosphate 3-kinase C), *Itpr1* (Inositol 1,4,5-trisphosphate receptor 1), *Oxtr* (Oxytocin receptor). TRFs: *Ahctf1* (AT hook containing transcription factor 1), *Arid3a* (AT rich interactive domain 3A (BRIGHT-like)), *Bhlha9* (Basic helix-loop-helix family, member a9), *Bnc2* (Basonuclin 2), *Camk1d* (Calcium/calmodulin-dependent protein kinase ID), *Cask* (Calcium/calmodulin-dependent serine protein kinase (MAGUK family)), *Cc2d1a* (Coiled-coil and C2 domain containing 1A), *Ccnh* (Cyclin H), *Cdk8* (Cyclin-dependent kinase 8), *Cno7* (CCR4-NOT transcription complex, subunit 7), *Ctcf* (CCCTC-binding factor), *Dpf1/2* (D4, zinc and double PHD fingers family 1/2), *Ercc2* (Excision repair cross-complementing rodent repair deficiency, complementation group 2), *Fos* (FBJ osteosarcoma oncogene), *Gabpb1* (GA repeat binding protein, beta 1), *Grhl3* (Grainyhead-like 3), *Gsk3b* (Glycogen synthase kinase 3 beta), *H2afy* (H2A histone family, member Y), *Hipk3* (Homeodomain interacting protein kinase 3), *Hnrnpd* (Heterogeneous nuclear ribonucleoprotein D), *Hoxb3/4/5* (Homeobox B3/4/5).

## Supplementary Materials

The following are available online at https://www.mdpi.com/2073-4425/10/10/754/s1, Table S1: REV values of the 93 quantified CAS genes in all stimulation paradigms, Table S2: REV values of the 934 quantified TRF genes in all stimulation paradigms.
